# Some remarks on b-(E.A)-property in b-metric spaces

**DOI:** 10.1186/s40064-016-2163-z

**Published:** 2016-04-27

**Authors:** Vildan Ozturk, Stojan Radenović

**Affiliations:** Department of Business Administration, Faculty of Economics and Administrative Science, Artvin Coruh University, 08000 Artvin, Turkey; Faculty of Mechanical Engineering, University of Belgrade, Kraljice Marije 16, Belgrad, 11120 Serbia; Department of Mathematics, State University of Novi Pazar, Novi Pazar, Serbia

**Keywords:** (E.A)-property, Common fixed point, Weakly compatible, b-Metric space, Well-posedness, 47H10, 54H25

## Abstract

In this paper we consider, discuss, improve and generalize recent b-(E.A)-property results for mappings in b-metric spaces established by Ozturk and Turkoglu (J Nonlinear Convex Anal 16(10):2059–2066, 2015). Thus, all our results are with much shorter proofs. One example is given to support the result.

## Introduction and preliminaries

Banach contraction principle (Banach 1922) was proved in 1922. After many generalizations of this principle were introduced in metric spaces. Also, in recent years some important generalizations of usual metric spaces were defined. *b*-metric space (Bakhtin 1989; Czerwik 1993) or metric type spaces-MTS by some authors (Hussain et al. 2012; Jovanović et al. 2010; Khamsi and Hussain 2010; Stanić et al. 2012) is well known generalizations of (usual) metric. Also several interesting results about the existence and uniqueness of fixed point were proved in *b*-metric spaces (Aghajani et al. 2014; Amini-Harandi 2014; Bakhtin 1989; Czerwik 1993; Ding et al. 2015a, b; Hussain et al. 2012, 2013; Jovanović et al. 2010; Khamsi and Hussain 2010; Kir and Kiziltunc 2013; Ozturk and Turkoglu 2015; Radenović and Kadelburg 2011; Roshan et al. 2013, 2014).

The following definition is introduced in Bakhtin (1989) and Czerwik (1993).

### **Definition 1**

(Bakhtin 1989; Czerwik 1993) Let *X* be a (nonempty) set and $s \ge 1$ be a given real number. A function $d:X\times X\rightarrow [0,\infty )$ is a *b*-metric on *X* if, for all $x,y,z\in X$, the following conditions hold:

1. $d\left( x,y\right) =0$ if and only if $x=y,$
2. $$d\left( x,y\right) =d\left( y,x\right) ,$$
3. $$d\left( x,z\right) \le s\left[ d\left( x,y\right) +d\left( y,z\right) \right]$$ (b-triangular inequality).

In this case, the pair (*X*, *d*) is called a *b*-metric space (metric type space).

It should be noted that, the class of *b*-metric spaces is effectively larger than that of metric spaces, every metric is a *b*-metric with $s=1,$ while the converse is not true.

### *Example 2*

Let (*X*, *d*) be a metric space and $\rho \left( x,y\right) =\left( d\left( x,y\right) \right) ^p$, where $p>1$ is a real number. Then $\rho$ is a *b*-metric with $s=2^{p-1}.$

However, if (*X*, *d*) is a metric space, then $\left( X,\rho \right)$ is not necessarily a metric space.

For example, if $X={\mathbb {R}}$ is the set of real numbers and $d\left( x,y\right) =\left| x-y\right|$ is usual Euclidean metric, then $\rho \left( x,y\right) =\left( x-y\right) ^2$ is a *b*-metric on ${\mathbb {R}}$ with $s=2.$ But is not a metric on ${\mathbb {R}}.$

### **Definition 3**

(Jovanović et al. 2010) Let $\left\{ x_n\right\}$ be a sequence in a *b*-metric space (*X*, *d*).

- $\left\{ x_n\right\}$ is called *b*-convergent if and only if there is $x\in X$ such that $d\left( x_n,x\right) \rightarrow 0$ as $n\rightarrow \infty .$
- $\left\{ x_n\right\}$ is a *b*-Cauchy sequence if and only if $d\left( x_n,x_m\right) \rightarrow 0$ as $n,m\rightarrow \infty .$

A *b*-metric space is said to be complete if and only if each *b*-Cauchy sequence in this space is *b*-convergent.

### **Proposition 4**

(Jovanović et al. 2010) *In ab*-*metric space*$\left( X,d\right) ,$*the following assertions hold*:

1. *Ab*-*convergent sequence has a unique limit*.
2. *Eachb*-*convergent sequence isb*-*Cauchy*.
3. *In general, ab*-*metric is not continuous*.

### **Definition 5**

(Hussain and Shah 2011) Let (*X*, *d*) be a *b*-metric space. A subset $Y\subset X$ is called closed if and only if for each sequence $\left\{ x_n\right\}$ in *Y* which *b*-converges to an element *x*,  we have $x\in Y.$

On the other hand (*E.A*)-property was introduced by Aamri and Moutawakil (2002). Later some authors introduced some new fixed point results using this concept (Ali et al. 2010; Babu and Sailaja 2011; Nazir and Abbas 2014; Ozturk and Turkoglu 2015). In this paper, we prove a common fixed point theorem for two pairs of mappings which satisfy the *b*-(*E.A*) property in *b*-metric spaces (Ozturk and Turkoglu 2015).

### **Definition 6**

Let (*X*, *d*) be a *b*-metric space and *f* and *g* be selfmappings on *X*.

(i) *f* and *g* are said to compatible if whenever a sequence $\left\{ x_{n}\right\}$ in *X* is such that $\left\{ fx_{n}\right\}$ and $\left\{ gx_{n}\right\}$ are *b*-convergent to some $t\in X,$ then
  $$\lim \nolimits _{n\rightarrow \infty }d\left( fgx_{n},gfx_{n}\right) =0.$$
(ii) *f* and *g* are said to noncompatible if there exists at least one sequence $\left\{ x_{n}\right\}$ in *X* is such that $\left\{ fx_{n}\right\}$ and $\left\{ gx_{n}\right\}$ are *b*-convergent to some $t\in X,$ but $\lim \nolimits _{n\rightarrow \infty }d\left( fgx_{n},gfx_{n}\right)$ is either nonzero or does not exist.
(iii) Ozturk and Turkoglu (2015)*f* and *g* are said to satisfy the *b*-(*E.A*) property if there exists a sequence $\left\{ x_{n}\right\}$ such that
  $$\lim \nolimits _{n\rightarrow \infty }fx_{n}=\lim \nolimits _{n\rightarrow \infty }gx_{n}=t,$$
  for some $$t\in X.$$

### *Remark 7*

Noncompatibility implies *b*-(*E.A*)-property.

### *Example 8*

Ozturk and Turkoglu (2015) Let $X=\left[ 0,2\right]$ and define $d:X\times X\rightarrow \left[ 0,\infty \right)$ as follows

$$d\left( x,y\right) =\left( x-y\right) ^{2}.$$

Let $f,g:X\rightarrow X$ be defined by

$$f\left( x\right) =\left\{ \begin{array}{ll} 1,&{} x\in \left[ 0,1\right] \\ \frac{x+1}{8},& x\in \left( 1,2\right] \end{array} \right. \quad g\left( x\right) =\left\{ \begin{array}{ll} \frac{3-x}{2},&{} \quad x\in \left[ 0,1\right] \\ \frac{x}{4}, &{} \quad x\in \left( 1,2\right] \end{array} \right. .$$

For a sequence $$\left\{ x_{n}\right\}$$ in *X* such that $$x_{n}=1+\frac{1}{ n+2},$$$$n=0,1,2,\ldots$$ and

$$\lim \nolimits _{n\rightarrow \infty }fx_{n}=\lim \nolimits _{n\rightarrow \infty }gx_{n}=\frac{1}{4}.$$

So *f* and *g* are satisfy the *b*-(*E.A*) property. $\lim \nolimits _{n\rightarrow \infty }d\left( fgx_{n},gfx_{n}\right)$ exists and it is not equal to 0. Thus *f* and *g* are noncompatible.

### **Definition 9**

(Jungck 1986) *f* and *g* be given self-mappings on a set *X*. The pair $\left( f,g\right)$ is said to be weakly compatible if *f* and *g* commute at their coincidence points (i.e. $fgx=gfx$ whenever $fx=gx$).

## Main results

In our first result of this section, we generalize, complement and improve recent results from (Ozturk and Turkoglu 2015, Theorems 2.1, Corollaries 2.2; 2.3 and Example 2.4) for b-metric spaces with much shorter proofs. We begin with our first result.

### **Theorem 10**

*Let*$\left( X,d\right)$*be a b-metric space with*$s>1$*and*$f,g,S,T:X\rightarrow X$*be a mappings with*$f\left( X\right) \subseteq T\left( X\right)$*and*$g\left( X\right) \subseteq S\left( X\right)$*such that*

1 $$s^{\,\,\varepsilon }d\left( fx,gy\right) \le M_s\left( x,y\right) ,\quad \textit{for}\, \textit{all}\,\,x,y\in X$$

*where*$\varepsilon >1$*is a constant and*

$$M_s\left( x,y\right) =\max \left\{ d\left( Sx,Ty\right) ,d\left( fx,Sx\right) ,d\left( gy,Ty\right) ,\frac{d\left( fx,Ty\right) +d\left( Sx,gy\right) }{2s}\right\}.$$

*Suppose that one of the pairs*$\left( f,S\right)$*and*$\left( g,T\right)$*satisfy the b*-(*E.A*)-*property and that one of the subspaces*$f\left( X\right) ,g\left( X\right) ,S\left( X\right)$*and*$T\left( X\right)$*is b-closed inX*.* Then the pairs*$\left( f,S\right)$*and*$\left( g,T\right)$*have a point of coincidence inX*.*Moreover, if the pairs*$\left( f,S\right)$*and*$\left( g,T\right)$*are weakly compatible, thenf*, *g*, *SandThave a unique common fixed point*.

### *Proof*

If the pair $\left( f,S\right)$ satisfies the *b*-(*E.A*)-property, then there exists a sequence $\left\{ x_{n}\right\}$ in *X* satisfying

$$\lim _{n\rightarrow \infty }fx_{n}=\lim _{n\rightarrow \infty }Sx_{n}=q,$$

for some $q\in X$. As $f\left( X\right) \subseteq T\left( X\right)$ there exists a sequence $\left\{ y_{n}\right\}$ in *X* such that $fx_{n}=Ty_{n}.$ Hence $\lim \nolimits _{n\rightarrow \infty }Ty_{n}=q.$ Let us show that $\lim \nolimits _{n\rightarrow \infty }gy_{n}=q.$ By (1),

2 $$s^{\,\varepsilon }d\left( fx_{n},gy_{n}\right) \le M_{s}\left( x_{n},y_{n}\right)$$

where

$$\begin{aligned} M_{s}\left( x_{n},y_{n}\right)= & \,\max \left\{ \begin{array}{c} d\left( Sx_{n},Ty_{n}\right) ,d\left( fx_{n},Sx_{n}\right) ,d\left( Ty_{n},gy_{n}\right) , \\ \frac{d\left( Sx_{n},gy_{n}\right) +d\left( fx_{n},Ty_{n}\right) }{2s} \end{array} \right\} \\= &\, \max \left\{ \begin{array}{c} d\left( Sx_{n},fx_{n}\right) ,d\left( fx_{n},Sx_{n}\right) ,d\left( fx_{n},gy_{n}\right) , \\ \frac{d\left( Sx_{n},gy_{n}\right) +d\left( fx_{n},fx_{n}\right) }{2s} \end{array} \right\} , \\ \le & \, \max \left\{ \begin{array}{c} d\left( Sx_{n},fx_{n}\right) ,d\left( fx_{n},gy_{n}\right) , \\ \frac{s\left[ d\left( Sx_{n},fx_{n}\right) +d\left( fx_{n},gy_{n}\right) \right] }{2s} \end{array} \right\} \\= & \, \max \left\{ d\left( Sx_{n},fx_{n}\right) ,d\left( fx_{n},gy_{n}\right) \right\} . \end{aligned}$$

In (2), on taking limit superior, we obtain

$$\begin{aligned} \mathop{\overline{\lim }}\limits_{n\rightarrow \infty }s^{\,\varepsilon }d\left( fx_{n},gy_{n}\right)\le & \, \mathop{\overline{\lim }}\limits_{n\rightarrow \infty }\max \left\{ d\left( Sx_{n},fx_{n}\right) ,d\left( fx_{n},gy_{n}\right) \right\} \\ \le &\, \mathop{\overline{\lim }}\limits_{n\rightarrow \infty }d\left( fx_{n},gy_{n}\right) , \end{aligned}$$

because $d\left( Sx_{n},fx_{n}\right) \rightarrow 0$ as $n\rightarrow \infty .$ Since $s^{\,\varepsilon }>s>1,$ we get that

$$\mathop{\overline{\lim}}\limits_{n\rightarrow \infty }d\left( fx_{n},gy_{n}\right) =0$$

that is $\mathop{\lim}\limits_{n\rightarrow \infty }d\left( fx_{n},gy_{n}\right) =0.$ Further, we have

$$\frac{1}{s}d\left( q,gy_{n}\right) \le d\left( q,fx_{n}\right) +d\left( fx_{n},gy_{n}\right) \rightarrow 0,$$

as $n\rightarrow \infty ,$ thus $gy_{n}\rightarrow q$ as $n\rightarrow \infty .$

If $T\left( X\right)$ is closed subspace of *X*,  then there exists a $r\in X,$ such that $Tr=q.$ We shall show that $gr=q.$ Indeed, we have

3 $$\begin{aligned} \frac{1}{s}d\left( q,gr\right)\le & \, d\left( q,fx_{n}\right) +d\left( fx_{n},gr\right) \nonumber \\ \le & \, d\left( q,fx_{n}\right) +\frac{1}{s^{\,\varepsilon }}M_{s}\left( x_{n},r\right) , \end{aligned}$$

where

$$\begin{aligned} M_{s}\left( x_{n},r\right)= & \,\max \left\{ \begin{array}{c} d\left( Sx_{n},Tr\right) ,d\left( fx_{n},Sx_{n}\right) ,d\left( Tr,gr\right) , \\ \frac{d\left( fx_{n},Tr\right) +d\left( Sx_{n},gr\right) }{2s} \end{array} \right\} \\= & \,\max \left\{ \begin{array}{c} d\left( Sx_{n},q\right) ,d\left( fx_{n},Sx_{n}\right) ,d\left( q,gr\right) , \\ \frac{d\left( fx_{n},q\right) +d\left( Sx_{n},gr\right) }{2s} \end{array} \right\} \\ \le & \, \max \left\{ \begin{array}{c} d\left( Sx_{n},q\right) ,d\left( fx_{n},Sx_{n}\right) ,d\left( q,gr\right) , \\ \frac{d\left( fx_{n},q\right) +sd\left( Sx_{n},q\right) +sd\left( q,gr\right) }{2s} \end{array} \right\} . \end{aligned}$$

Letting $n\rightarrow \infty ,$ we have

$$\lim _{n\rightarrow \infty }M_{s}\left( x_{n},r\right) \le \max \left\{ 0,0,d\left( q,gr\right) ,\frac{d\left( q,gr\right) }{2}\right\} =d\left( q,gr\right).$$

Now, (3) implies

$$\frac{1}{s}d\left( q,gr\right) \le 0+\frac{1}{s^{\,\varepsilon }}d\left( q,gr\right),$$

from which it follows $d\left( q,gr\right) =0$ (because $s^{\,\varepsilon }>s$). Hence, $q=gr=Tr,$ that is *r* is the coincidence point of pair $\left( g,T\right) .$ As $g\left( X\right) \subseteq S\left( X\right) ,$ there exists a point $z\in X$ such that $q=Sz.$ We claim that $Sz=fz.$ By (1), we have

4 $$s^{\,\varepsilon }d\left( fz,gr\right) \le M_{s}\left( z,r\right)$$

where

$$\begin{aligned} M_{s}\left( z,r\right) &= \max \left\{ d\left( Sz,Tr\right) ,d\left( fz,Sz\right) ,d\left( Tr,gr\right) ,\frac{d\left( fz,Tr\right) +d\left( Sz,gr\right) }{2s}\right\} \\ &= \max \left\{ 0,d\left( fz,q\right) ,0,\frac{d\left( fz,q\right) +0}{2s} \right\} \\& = d\left( fz,q\right) . \end{aligned}$$

Thus from (4),

$$s^{\,\varepsilon }d\left( fz,gr\right) \le d\left( fz,q\right) =d\left( fz,gr\right),$$

from which it follows that $d\left( fz,gr\right) =0.$ Therefore $Sz=fz=q.$ Hence *z* is the coincidence point of the pair $\left( f,S\right) .$ Thus $fz=Sz=gr=Tr=q.$ By the weak compatibility of the pairs $\left( f,S\right)$ and $\left( g,T\right) ,$ we btain that $fq=Sq$ and $gq=Tq.$

We will show that *q* is a common fixed point of *f*, *g*, *S* and *T*. From (1) we have

5 $$s^{\,\varepsilon }d\left( fq,q\right) =s^{\,\varepsilon }d\left( fq,gr\right) \le M_s\left( q,r\right)$$

where,

$$\begin{aligned} M_s\left( q,r\right)&= {} \max \left\{ d\left( Sq,Tr\right) ,d\left( fq,Sq\right) ,d\left( Tr,gr\right) ,\frac{d\left( fq,Tr\right) +d\left( Sq,gr\right) }{2s}\right\} \\ &= \max \left\{ d\left( fq,q\right) ,d\left( fq,fq\right) ,d\left( q,q\right) ,\frac{d\left( fq,q\right) +d\left( fq,q\right) }{2s}\right\} \\ &= d\left( fq,q\right) . \end{aligned}$$

By (5)

$$s^{\,\varepsilon }d\left( fq,q\right) \le d\left( fq,q\right),$$

from which it follows $fq=Sq=q.$ Similarly, it can be shown $gq=Tq=q.$

To prove the uniqueness of fixed point, suppose that *p* is another fixed point of *f*, *g*, *S* ad *T*. By (1),

$$s^{\,\varepsilon }d\left( q,p\right) =s^{\,\varepsilon }d\left( fq,gp\right) \le M_{s}\left( q,p\right)$$

where

$$\begin{aligned} M_{s}\left( q,p\right)= & \, \max \left\{ d\left( Sq,Tp\right) ,d\left( fq,Sq\right) ,d\left( Tp,gp\right) ,\frac{d\left( fq,Tp\right) +d\left( Sq,gp\right) }{2s}\right\} \\= & \, \max \left\{ d\left( q,p\right) ,d\left( q,q\right) ,d\left( p,p\right) , \frac{d\left( q,p\right) +d\left( q,p\right) }{2s}\right\} \\= & \, d\left( q,p\right) . \end{aligned}$$

Hence, we have

$$s^{\,\varepsilon }d\left( q,p\right) \le d\left( q,p\right),$$

from which again follows that $$d\left( q,p\right) =0.$$ So $$q=p.$$$$\square$$

### *Remark 11*

Since the condition (1) from Ozturk and Turkoglu (2015) implies (1) for all altering functions $\psi$ and $\varphi$ (Khan et al. 1984; Radenović et al. 2012) and for each $\varepsilon \in (1,2]$ we get that our Theorem 9 is genuine generalization of the main result from Ozturk and Turkoglu (2015). However, the main result from Ozturk and Turkoglu (2015) holds if $s=1,$ what is not case for our Theorem 9. Also, the main result from Ozturk and Turkoglu (2015) holds if $s>1$ and $\varepsilon \in \left( 1,\infty \right)$ for given altering functions $\psi$ and $\varphi .$

### **Corollary 12**

*Let*$\left( X,d\right)$*be a b-metric space and*$f,T:X\rightarrow X$*be mappings such that*

6 $$s^{\,\varepsilon }d\left( fx,fy\right) )\le M_s\left( x,y\right) \quad for\, all\,\, x,y\in X,$$

*where*$\varepsilon >1$*is a constant and*

$$M_s\left( x,y\right) =\max \left\{ d\left( Tx,Ty\right) ,d\left( fx,Tx\right) ,d\left( fy,Ty\right) ,\frac{d\left( fx,Ty\right) +d\left( Tx,fy\right) }{2s}\right\},$$

*Suppose that the pair*$\left( f,T\right)$*satisfies the b*-$(\textit{E.A})$-*property andT*(*X*) *is closed inX*. *Then the pair*$\left( f,T\right)$*has a unique point of coincidence inX*. *Moreover, if the pair*$\left( f,T\right)$*is weakly compatible, thenfandThave a unique common fixed point*.

### **Corollary 13**

*Let*$\left( X,d\right)$*be ab*-*metric space and*$f,T:X\rightarrow X$*be mappings such that*

7 $$s^{\,2}d\left( fx,fy\right) )\le M_s\left( x,y\right) \quad for\, all\,\, x,y\in X,$$

*where*$\varepsilon >1$*is a constant and*

$$M_s\left( x,y\right) =\max \left\{ d\left( Tx,Ty\right) ,d\left( fx,Tx\right) ,d\left( fy,Ty\right) ,\frac{d\left( fx,Ty\right) +d\left( Tx,fy\right) }{2s}\right\},$$

*Suppose that the pair*$\left( f,T\right)$*satisfies the b*-(*E.A*)-*property and*$T\left( X\right)$*is closed inX*. *Then the pair*$\left( f,T\right)$*has a unique point of coincidence inX*. *Moreover, if the pair*$\left( f,T\right)$*is weakly compatible, thenfandThave a unique common fixed point*.

### *Example 14*

Let $X=\left[ 0,1\right]$ and define $d:X\times X\rightarrow \left[ 0,\infty \right)$ as follows

$$\begin{aligned} d\left( x,y\right) =\left\{ \begin{array}{ll} 0,&{}\quad x=y \\ \left( x+y\right) ^{2},&{}\quad x\ne y \end{array} \right. . \end{aligned}$$

Then $\left( X,d\right)$ is a *b*-metric space with $s=2.$ Let $f,g,S,T:X\rightarrow X$ be defined by

$$f\left( x\right) =\frac{x}{4}\quad S\left( x\right) =\left\{ \begin{array}{ll} 2x,& 0\le x<\frac{1}{2} \\ 1,& x=\frac{1}{2}\\ \frac{1}{8}, & \frac{1}{2}<x\le 1 \end{array} \right.,\quad g\left( x\right) =0\ \quad \text { and }T\left( x\right) =x.$$

Clearly, $$g\left( X\right)$$ is closed, $$f\left( X\right) \subseteq T\left( X\right)$$ and $$g\left( X\right) \subseteq S\left( X\right)$$. The sequence $$\left\{ x_{n}\right\} ,$$$$x_{n}=\frac{1}{2}+\frac{1}{n}$$, is in *X* such that $$\lim \nolimits _{n\rightarrow \infty }fx_{n}=\lim \nolimits _{n\rightarrow \infty }Sx_{n}=\frac{1}{8}.$$ So that the pair $$\left( f,S\right)$$ satisfies the *b*-(*E.A*)-property. But the pair $$\left( f,S\right)$$ is noncompatible because $$\lim \nolimits _{n\rightarrow \infty }d\left( fSx_{n},Sfx_{n}\right) \ne 0.$$ To check the contractive condition (1), for all $$x,y\in X,$$ and $$\varepsilon =2,$$

if $x=0,$ then (1) is satisfied.

If $$x\in \left( 0,\frac{1}{2}\right)$$, then

$$\begin{aligned} s^{\,\varepsilon }d\left( fx,gy\right) =2^{\,2}\cdot \left( \frac{x}{4} \right) ^{2}\le \left( \frac{9x}{4}\right) ^{2}=d\left( fx,Sx\right) \le M_{s}\left( x,y\right) . \end{aligned}$$

If $$x=\frac{1}{2},$$ then

$$\begin{aligned} s^{\,\varepsilon }d\left( fx,gy\right) =2^{\,2}\cdot \left( \frac{1}{8} \right) ^{2}\le \left( \frac{1}{8}+1\right) ^{2}=d\left( fx,Sx\right) \le M_{s}\left( x,y\right) . \end{aligned}$$

If $$x\in \left( \frac{1}{2},1\right]$$, then

$$\begin{aligned} s^{\,\varepsilon }d\left( fx,gy\right) =2^{\,2}\cdot \left( \frac{x}{4} \right) ^{2}\le \left( \frac{x}{4}+\frac{1}{8}\right) ^{2}=d\left( fx,Sx\right) \le M_{s}\left( x,y\right) . \end{aligned}$$

Thus (1) is satisfied for all $x,y\in X.$ The pairs (*f*; *S*) and (*g*; *T*) are weakly compatible. Hence, all the conditions of Theorem 9 are satisfied. Moreover 0 is the unique common fixed point of *f*; *g*; *S* and *T*.

The following results are similar to previously and the proofs are omitted.

### **Theorem 15**

*Let*$\left( X,d\right)$*be a b-metric space with*$s>1$*and*$f,g,S,T:X\rightarrow X$*be a mappings with*$f\left( X\right) \subseteq T\left( X\right)$*and*$g\left( X\right) \subseteq S\left( X\right)$*such that*

8 $$\begin{aligned} s^{\,\,\varepsilon }d\left( fx,gy\right) \le M_{s}\left( x,y\right) ,\quad \textit { for all }x,y\in X \end{aligned}$$

*where*$\varepsilon >1$*is a constant and*

$$\begin{aligned} M_{s}\left( x,y\right) =\max \left\{ d\left( Sx,Ty\right) ,\frac{d\left( fx,Sx\right) +d\left( gy,Ty\right) }{2s},\frac{d\left( fx,Ty\right) +d\left( Sx,gy\right) }{2s}\right\} . \end{aligned}$$

*Suppose that one of the pairs*$\left( f,S\right)$*and*$\left( g,T\right)$*satisfy the b*-(*E.A*)-*property and that one of the subspaces*$f\left( X\right) ,g\left( X\right) ,S\left( X\right)$*and*$T\left( X\right)$*is b-closed inX*. *Then the pairs*$\left( f,S\right)$*and*$\left( g,T\right)$*have a point of coincidence inX*.* Moreover, if the pairs*$\left( f,S\right)$*and*$\left( g,T\right)$*are weakly compatible, thenf*, *g*, *SandThave a unique common fixed point*.

### **Theorem 16**

*Let*$\left( X,d\right)$*be ab*-*metric space with*$s>1$*and*$f,g,S,T:X\rightarrow X$*be a mappings with*$f\left( X\right) \subseteq T\left( X\right)$*and*$g\left( X\right) \subseteq S\left( X\right)$*such that*

9 $$s^{\,\,\varepsilon }d\left( fx,gy\right) \le M_{s}\left( x,y\right) ,\quad for\, all\,\, x,y\in X$$

where $\varepsilon >1$*is a constant and*

$$\begin{aligned} M_{s}\left( x,y\right) =\max \left\{ d\left( Sx,Ty\right) ,d\left( fx,Sx\right) ,d\left( gy,Ty\right) ,d\left( fx,Ty\right) ,d\left( Sx,gy\right) \right\} . \end{aligned}$$

*Suppose that one of the pairs*$\left( f,S\right)$*and*$\left( g,T\right)$*satisfy the b*-(*E.A*)-*property and that one of the subspaces*$f\left( X\right) ,g\left( X\right) ,S\left( X\right)$*and*$T\left( X\right)$*is b-closed inX*. *Then the pairs*$\left( f,S\right)$*and*$\left( g,T\right)$*have a point of coincidence inX*. *Moreover, if the pairs*$\left( f,S\right)$*and*$\left( g,T\right)$*are weakly compatible, thenf*, *g*, *SandThave a unique common fixed point*.

## Well posedness result

### **Definition 17**

(Boriceanu et al. 2010) Let $\left( X,d\right)$ be a *b*-metric space and $f:X\rightarrow X$ be a mapping. The fixed point problem of *f* said to be well-posed iff

(i) *f* has a unique fixed point *q* in *X*, 
(ii) If $$x_{n}\in X,$$$$n\in {\mathbb {N}}$$ and $$d\left( x_{n},Tx_{n}\right) \rightarrow 0$$ as $$n\rightarrow \infty ,$$ then $$d\left( x_{n},q\right) \rightarrow 0$$ as $$n\rightarrow \infty .$$

### **Theorem 18**

*Let*$\left( X,d\right)$*be ab*-*metric space with*$s>1$*and*$f,g,S,T:X\rightarrow X$*be a mappings. Suppose that all hypotheses of Theorem 9 hold. Then the fixed point problem is well posed for mappingsf*, *g*, *SandT*.

### *Proof*

By Theorem 9, The mappings *f*, *g*, *S* and *T* have a unique common fixed point (say) *q* in *X*.Let $\left\{ x_{n}\right\}$ be a sequence in *X* such that

$$\begin{aligned} \mathop{\lim}\limits _{n\rightarrow \infty }d\left( fx_{n},x_{n}\right) &= \mathop{\lim}\limits _{n\rightarrow \infty }d\left( gx_{n},x_{n}\right) =\lim \nolimits _{n\rightarrow \infty }d\left( Tx_{n},x_{n}\right) \\& = \mathop{\lim}\limits _{n\rightarrow \infty }d\left( Sx_{n},x_{n}\right) =0. \end{aligned}$$

Using (1) and triangular inequality, we have

$$\begin{aligned} d\left( q,x_{n}\right) &= d\left( fq,x_{n}\right) \le s\left[ d\left( fq,gx_{n}\right) +d\left( gx_{n},x_{n}\right) \right] \\&= \frac{s}{s^{\varepsilon }}M_{s}\left( q,x_{n}\right) +sd\left( gx_{n},x_{n}\right) \end{aligned}$$

where

$$\begin{aligned} M_{s}\left( q,x_{n}\right)= & \, \max \left\{ \begin{array}{c} d\left( Sq,Tx_{n}\right) ,d\left( fq,Sq\right) ,d\left( gx_{n},Tx_{n}\right) , \\ \frac{d\left( fq,Tx_{n}\right) +d\left( Sq,gx_{n}\right) }{2s} \end{array} \right\} \\= & {} \max \left\{ d\left( q,Tx_{n}\right) ,0,d\left( gx_{n},Tx_{n}\right) , \frac{d\left( q,Tx_{n}\right) +d\left( q,gx_{n}\right) }{2s}\right\} \\ \le & \, \max \left\{ \begin{array}{c} d\left( q,Tx_{n}\right) ,s\left[ d\left( gx_{n},q\right) +d\left( q,Tx_{n}\right) \right] , \\ \frac{d\left( q,Tx_{n}\right) +d\left( q,gx_{n}\right) }{2s} \end{array} \right\} \\= & \, s\left[ d\left( gx_{n},q\right) +d\left( q,Tx_{n}\right) \right] . \end{aligned}$$

Thus we have

$$d\left( q,x_{n}\right) \le \frac{s^{2}}{s^{\varepsilon }}\left[ d\left( gx_{n},q\right) +d\left( q,Tx_{n}\right) \right] +sd\left( gx_{n},x_{n}\right).$$

By triangular inequality,

$$d\left( q,x_{n}\right) \le \frac{s^{3}}{s^{\varepsilon }}\left[ d\left( gx_{n},x_{n}\right) +2d\left( x_{n},q\right) +d\left( x_{n},Tx_{n}\right) \right] +sd\left( gx_{n},x_{n}\right).$$

So

$$d\left( q,x_{n}\right) \le \frac{1}{\left( 1-\frac{2s^{3}}{s^{\varepsilon }} \right) }\left[ \left( \frac{s^{3}}{s^{\varepsilon }}+s\right) d\left( gx_{n},x_{n}\right) +\frac{s^{3}}{s^{\varepsilon }}d\left( x_{n},Tx_{n}\right) \right].$$

On taking the limit as $n\rightarrow \infty$ we obtain

$$\lim \nolimits _{n\rightarrow \infty }d\left( q,x_{n}\right) =0.$$

$$\square$$

## Conclusion

In this paper, we given new fixed point theorems for mappings satisfying *b*-(*E.A*)-property in *b*-metric spaces. Our results extended *b*-(*E.A*)-property results in the literature. Also in last section we proved well-posedness result in *b*-metric spaces.
